# A Variable Data Fusion Approach for Electromechanical Impedance-Based Damage Detection

**DOI:** 10.3390/s20154204

**Published:** 2020-07-28

**Authors:** Shishir Kumar Singh, Rohan Soman, Tomasz Wandowski, Pawel Malinowski

**Affiliations:** Institute of Fluid Flow Machinery, Polish Academy of Sciences, 80-231 Gdansk, Poland; shishir85mech@imp.gda.pl (S.K.S.); tomaszw@imp.gda.pl (T.W.); pmalinowski@imp.gda.pl (P.M.)

**Keywords:** electromechanical impedance, data fusion, damage severity, damage detection

## Abstract

There is continuing research in the area of structural health monitoring (SHM) as it may allow a reduction in maintenance costs as well as lifetime extension. The search for a low-cost health monitoring system that is able to detect small levels of damage is still on-going. The present study is one more step in this direction. This paper describes a data fusion technique by combining the information for robust damage detection using the electromechanical impedance (EMI) method. The EMI method is commonly used for damage detection due to its sensitivity to low levels of damage. In this paper, the information of resistance (R) and conductance (G) is studied in a selected frequency band and a novel data fusion approach is proposed. A novel fused parameter (F) is developed by combining the information from G and R. The difference in the new metric under different damage conditions is then quantified using established indices such as the root mean square deviation (RMSD) index, mean absolute percentage deviation (MAPD), and root mean square deviation using *k*-th state as the reference (RMSDk). The paper presents an application of the new metric for detection of damage in three structures, namely, a thin aluminum (Al) plate with increasing damage severity (simulated with a drilled hole of increasing size), a glass fiber reinforced polymer (GFRP) composite beam with increasing delamination and another GFRP plate with impact-induced damage scenarios. Based on the experimental results, it is apparent that the variable F increases the robustness of the damage detection as compared to the quantities R and G.

## 1. Introduction

All man-made civil, aerospace and mechanical structures have a limited lifespan and are prone to structural defects like corrosion, fatigue, wear, delamination, etc. These structural defects can be monitored using suitable SHM techniques. Electromechanical Impedance (EMI) method is one of the SHM techniques that have been used in high-frequency ranges to assess the local health of a structure [1,2]. The growing research trend in EMI based detection is on the development of the data-driven approach to accurately detect location and severity of structural defects [3,4]. Ai et al. [5] used effective structural mechanical impedance in damage detection in reinforced concrete (RC). They perform a comparative study of the effectiveness of electromechanical admittance, structural mechanical impedance and effective structural mechanical impedance signature for damage detection. Further work was done by Zuo et al. using multiple sensors for crack identification in the pipeline system. They derived the damage sensitive feature from the active part of the raw admittance [6]. Recently, Zhu et al. proposed a comprehensive active monitoring scheme direct coupling mechanical impedance-based signature extraction using a modified electromechanical impedance analytical model. They used root mean square difference (RMSD) index to locate and quantify the extent of the disbond inside the structures [7,8]. Apart from developments in the EMI-based damage detection, data fusion-based techniques, too, are receiving a renewed interest in the last few years. Zhao et al. [9] proposed the hierarchical ensemble scheme to data fusion based on Dempster–Shafer (DS) theory and the Rotation Forest (RF) method. RF was used to build an accurate and diverse base and DS was used to combine the output of RF data sources [9,10]. Feature level fusion-based machine learning techniques like artificial neural network (ANN), support vector machine (SVM) and deep learning are becoming popular in data fusion [11]. Recently, Chen proposed deep learning-based data fusion concept to detect and localize cracks on the metallic surfaces of the nuclear power plant’s reactors [12]. The use of principal component analysis (PCA) for damage detection and classification is receiving attention as well. Quin et al. used PCA based Q statistics and T^2^ statistics in fault detection and diagnosis of polyester film manufacturing process [13]. Mujica explored these statistical techniques to detect and distinguish damage in steel plate and turbine blade structures [14]. Tibaduiza proposed a data-driven statistical approach using PCA for damage classification for distributed piezoelectric active sensor network for time-domain vibrational structural responses [15]. Park et al. employed the PCA model for impedance data in identifying loose bolts in bolted aluminum plate structure in wireless SHM [16]. They used an on-board active sensor system consisting of impedance measuring chips and macro-fiber composite sensors. Although data fusion for EMI is a newly trending approach, the framework for data fusion is well established in other fields of study. Data fusion is defined as merging information into a new set of information for reducing the uncertainty and allowing more efficient representation. Data fusion can be done at three levels: the data level fusion, decision-level fusion and feature-level fusion. The data level data fusion allows pure data fusion as it avoids the propagation and magnification of the noise and measurement errors due to post-processing. It also yields a better signal to noise ratio [17,18,19] and hence is investigated in this paper. The use of data fusion is inspired by a need to obtain a unified approach of damage detection for the structure. The data fusion allows the extraction of information from frequency domain data to improve damage detection through robust decision making. This paper develops a low-level sensor data fusion based damage detection using EMI technique. Although significant work has been done on data fusion, very few variable data fusion techniques have been implemented for EMI based SHM technique and as such is the novelty of the paper. In this paper, variable data fusion in EMI technique is implemented for damage detection of isotropic and anisotropic material (aluminum and composite plates). The data fusion is achieved through the development of the fused parameter (F) by combining the resistance (R) and conductance (G) of the measured impedance spectra. The RMSD, root mean square difference using kth state as the reference (RMSDk) and mean absolute percentage deviation (MAPD) damage indices are used for quantifying the difference in the fused parameter F. The paper presents the proof of concept of the fused variable and the improved performance for damage detection as compared to the resistance and conductance-based results.

## 2. Theory and Methodology

The electrical impedance of the bonded piezo electric transducer (PZT) is equal to the voltage (V) applied to the PZT divided by the current passing through it. The measured electric current (I) is used to calculate the EMI (Z(ω)) [8,20]:

(1) $$\begin{array}{c} Z\left(\omega \right)=\frac{V}{I}=R+jX=\frac{1}{Y}=\frac{1}{\left(G+jB\right)}\\ =\frac{1}{j\omega }\frac{h_{p}}{{\bar{\epsilon }}_{33}^{T}\pi a^{2}}{\left[1-\frac{2d_{31}^{2}}{{\bar{s}}_{11}^{E}{\bar{\epsilon }}_{33}^{T}\left(1-\nu \right)}\left[1-\frac{1}{\frac{Z_{a}\left(\omega \right)}{Z_{s}\left(\omega \right)}+1}\left(\frac{2}{\bar{\phi }}\right)\left(\frac{J_{1}\left(\bar{\phi }\right)}{J_{0}\left(\bar{\phi }\right)}\right)\right]\right]}^{-1}, \end{array}$$

where $\bar{\phi }=\frac{\omega }{\bar{c_{p}}}a$, $\bar{c_{p}}=\frac{1}{\sqrt{\rho {\bar{s}}_{11}^{E}\left(1-\nu^{2}\right)}}$, ${\bar{s}}_{11}^{E}=s_{11}^{E}\left(1-j\eta \right)$, ${\bar{\epsilon }}_{33}^{T}=\epsilon_{33}^{T}\left(1-j\delta \right)$, $J_{0}$ and $J_{1}$ are zero and first order Bessel function of first kind respectively, $Z_{a}$ is the short-circuited mechanical impedance of piezoelectric transducer (actuator), $Z_{s}$ is the mechanical impedance of structure, $h_{p}$ is the thickness of piezoelectric transducer, *a* is the radius of transducer, ρ is the density, $s_{11}^{E}$ is compliance coefficient, $d_{31}$ is the piezoelectric coefficient of transducer for direction 3–1 (electric field applied in direction 3, strains in direction 1), *j* is the complex symbol, $\epsilon_{33}$ is the complex permittivity of piezoelectric transducer for direction 33, η is the mechanical loss factor and δ is dielectric loss factor.

The R and G are EMI parameters that in many applications were found very effective for damage detection in the structure. The relation between R and G can be determined from the definition of electrical impedance (Z) and admittance (Y):

(2) $$Z=R+jX=\frac{1}{Y}=\frac{1}{\left(G+jB\right)}=\frac{1}{\left(G+jB\right)}\times \frac{\left(G-jB\right)}{\left(G-jB\right)}=\frac{\left(G-jB\right)}{\left(G^{2}+B^{2}\right)}=\frac{\left(G-jB\right)}{{\left[|Y|\right]}^{2}},$$

where *X* is the reactance and *B* is the susceptance. of the system. After comparing the real and imaginary parts of Equation (2) the relation of *R*, *G*, *B*, and *X* can be found:

(3) $$R=\frac{G}{G^{2}+B^{2}};\;X=\frac{-B}{G^{2}+B^{2}};\;G=\frac{R}{R^{2}+X^{2}};\;B=\frac{-X}{R^{2}+X^{2}}.$$

Multiplying *G* by *R* we define a new fused non-dimensional parameter which is a function of two quantities (G and R, or G and B, or R and X—Equation (4)). This multiplicative dimensionless parameter may be used for the damage detection.

(4) $$F=G\times R=\frac{G^{2}}{G^{2}+B^{2}}=\frac{R^{2}}{R^{2}+X^{2}}.$$

The F amplifies the common peaks of G and R and hence is expected to improve the damage detection capabilities. Researchers up to now chose only one quantity for the EMI based damage assessment in a relatively narrow frequency band. For instance, Annamdas et al. [21] focused on conductance and limited themselves to a range of 40–160 kHz, further narrowing the conductance curves analysis to narrower ranges. Perera et al. [22] and Baptista et al. [23] underlined the better damage sensitivity of resistance for a narrow frequency band namely 10–80 kHz range for the aluminum sample with bolted joint and 16–40 kHz for the aluminum beam with damage modeled by additional mass. The low-frequency range 10–80 kHz for resistance was also used by Na et al. [24] for carbon composites. So in the proposed methodology, by the combination of the two variables, the sensitivity of the variable is leveraged to a more intermediate sensitivity over a larger frequency range. As a result, the indices based on the variable F are more robust for damage assessment and can be applied over a larger frequency range. The work was loosely motivated by the work by Soman et al [25]. In their work on vibration based damage detection in structures, two variables with higher sensitivities in a small domain are combined to form a variable with an intermediate sensitivity over the entire domain.

The damage detection using the EMI technique is based on quantifying the difference in the measurement of the structure at a given point of time with a known previous condition. RMSD and MAPD are the most popular damage detection indices employed for this quantification. Equations (5) and (6) are used to quantify damage with respect to a healthy state of the structure in the EMI techniques [26,27]:

(5) $$RMSD=\sqrt{\frac{\sum_{i=1}^{n}{\left(D_{i}-D_{i}^{o}\right)}^{2}}{\sum_{i=1}^{n}{\left(D_{i}^{o}\right)}^{2}}}$$

(6) $$MAPD=100/n\times \sum_{i=1}^{n}\left|\left(D_{i}-D_{i}^{o}\right)/\left(D_{i}^{o}\right)\right|.$$

The recent use of RMSDk improves the damage detection performance significantly [5]. This paper also investigates damage index RMSDk using these parameters, which is used to differentiate and evaluate the absolute variations of damage severity at each level. Equation (7) gives the formula for calculating the RMSDk. The key difference in the RMSD and RMSDk is that for the RMSDk the measured data is compared with the previous state rather than the healthy state.

(7) $$RMSDk=\sqrt{\frac{\sum_{i=1}^{n}{\left(D_{i}-D_{i}^{k}\right)}^{2}}{\sum_{i=1}^{n}{\left(D_{i-1}\right)}^{2}}},$$

where the symbol *n* is used for the number of frequency spectrum samples, k is used for *k*-th state, $D_{i}$ is the *i*-th damage state. $D_{i-1}$ is the single sample of the spectrum divided by frequency for the single sample of the spectrum divided by frequency for (i−1)-th damage state. It is known that *G* and *R* show a trend with the frequency, so the measurements were rescaled (detrended) by multiplying the values by the normalized frequency for each measurement of R and dividing by the normalized frequency for each measurement of G as shown by the Equation (8). The above operation maintains the units of *G*, *R* and *F*.

(8) $$G_{rescaled}=\frac{G\times f_{max}}{f};\;R_{rescaled}=\frac{R\times f}{f_{max}};\;F_{rescaled}=\frac{F\times f_{max}}{f}.$$

**Figure 1:**
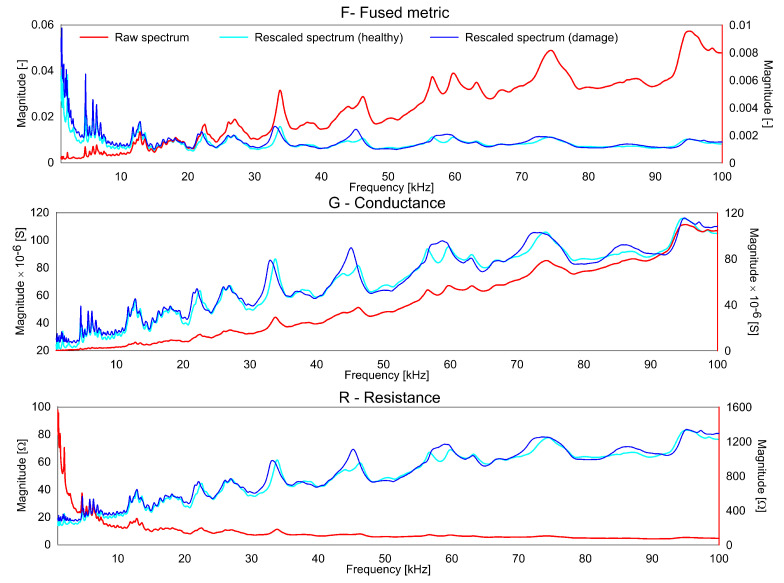
Raw and rescaled spectrum (healthy and damage scenario) for F, G, R.

It should be noted, that the rescaling operation for EMI measurements for R and G was used before [28]. For the variable F, the decision for the rescaling was made based on the correlation to the frequency observed as shown in Figure 1. For the purpose of conciseness, $F_{rescaled}$, $G_{rescaled}$ and $R_{rescaled}$ will be referred to as *F*, *G* and *R* from here on. As can be seen, the F plot is considerably flattened by the rescaling. Similarly, the G plot has more peaks obvious in the lower frequency range due to the rescaling, the contributions of the lower frequencies is reduced for the R plot.

The rescaled spectra for G, R and F for healthy and one damage scenario are shown in Figure 1. As can be seen, the F spectra is smoother than the G and R spectra. The computation of F minimizes the effect of the non-dominant peaks which are often related to the measurement noise and other uncertainties. As the contribution of these uncertainties to the index for quantifying the difference is reduced, the F yields more stable results. At the same time, the F amplifies the common peaks in the G and R giving a higher contribution to these common peaks.

## 3. Experimental Setup and Objects of Investigation

The experiments were performed on both metal and composites with attached piezo-actuators for validation of the proposed methodology. The EMI measurements were conducted using Ceramtec piezoelectric transducers made of SONOX P502 material with a disc shape of 0.5 mm thickness and 10 mm diameter. In the research, HIOKI IM 3570 analyzer used for the measurements. The sensors are glued on the metal and composites using cyanoacrylate glue and soldered wires on the top of the sample surfaces connected to the impedance analyzer. The chirp excitation was used for the study with an amplitude of 1 V. The locations for damage simulation were chosen based on engineering judgment ensuring that the chosen damage locations lie in the sensitivity range of the sensor of interest. The three structures studied were:

1. A small aluminum plate with drilled holes.
2. The glass fiber reinforced polymer (GFRP) beam with delamination.
3. The GFRP plate with several impacts on its surface.

**Figure 2:**
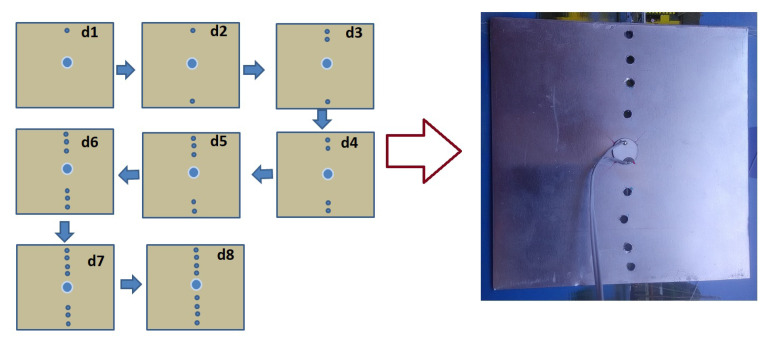
The sequences of holes creation and the created hole on Al square plate.

For the first study, a square aluminum plate with dimensions 100 mm × 100 mm × 1 mm was used. Holes were introduced sequentially from either side of the plate (top and bottom) towards the center of the PZT. The diameter of each hole is 3 mm. The distance between the holes is about 1 cm. The nearest holes are at a distance of 1.5 cm from the center of the piezo-actuator. Two sets of measurements were carried out for the reference (healthy state) and after each hole was introduced in the plate. The measurements were carried out at room temperature (23 ± 1${}^{\circ }$). The diagram of drilled holes and their sequence are given in Figure 2. The measurements were carried out over the frequency range of 1 kHz to 5 MHz with a step size of 200 Hz.

**Figure 3:**
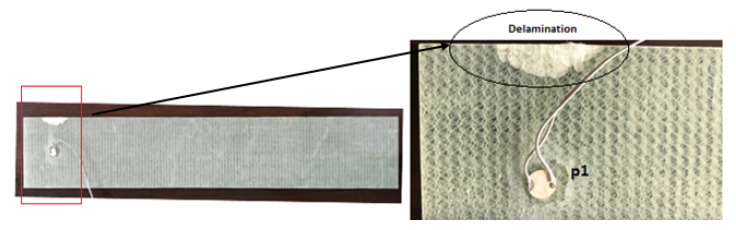
Composite beam with enlarged view showing delaminated region (S3).

The second set of experiments were performed on a rectangular thick composite beam (500 mm × 95 mm × 3 mm) with a single piezoelectric transducer attached as shown in the Figure 3. EMI responses were acquired from the composite plate in a healthy state and three damaged states (with increasing delamination). The scalpel was used for introducing the simulated delamination. The dimensions of delamination were: (S1) 10 mm × 5mm, (S2) 20 mm × 10 mm, and (S3) 30 mm × 10 mm. For each stage of the delamination, measurements were taken twice. The measurements were carried out over the frequency range of 1 kHz to 100 kHz with a step size of 10 Hz.

**Figure 4:**
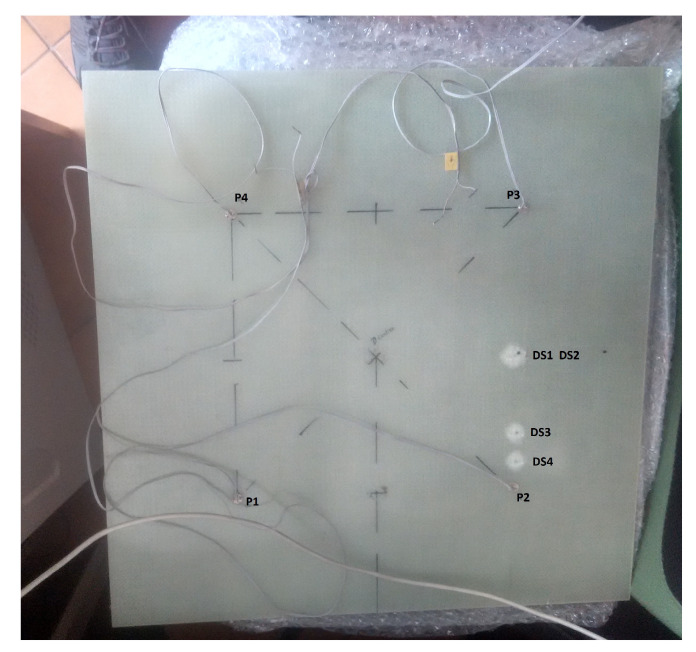
The glass fiber reinforced polymer (GFRP) plate with impact locations.

The third sample studied was a woven GFRP plate (Figure 4) of the size 500 mm × 500 mm × 3 mm. The measurements were performed in stable room temperature conditions and in the frequency range of 1 kHz to 5 MHz with a step of 200 Hz. The impact was made by dropping a projectile with a spherical end on the sample surface. The impact was made by a steel ball integrated with a cuboid structure having a total mass of 1.3 kg, the height for dropping the projectile was 2.39 m. The estimated energy of a single impact was 30 J. The total four impact made on sample and description of the impact data on the GFRP sample surface is given in Table 1. Although the figure shows 4 PZT sensors, only results from sensor P2 are presented here. The other sensors were far away from the impact locations and were observed to be insensitive to damage.

The three structures studied were part of different studies and hence the frequency ranges and the step size for the measurements were different. However, in order to maintain consistency in the measurements and allow a comparison for all three structures, only the frequency range of 1 kHz to 100 kHz was analyzed and presented here. Another point to note is that each measurement carried out is an averaged value of 50 measurements carried out at each frequency and hence are considered statistically stable. The changes seen in two measurements for the same scenario are due to the different ambient conditions. These changes in different measurements are accounted for with the threshold value which is defined based on the difference in the measurements for two measurements in the known healthy condition.

## 4. Results and Discussions

### 4.1. Damage Detection in Aluminium Plate

**Figure 5:**
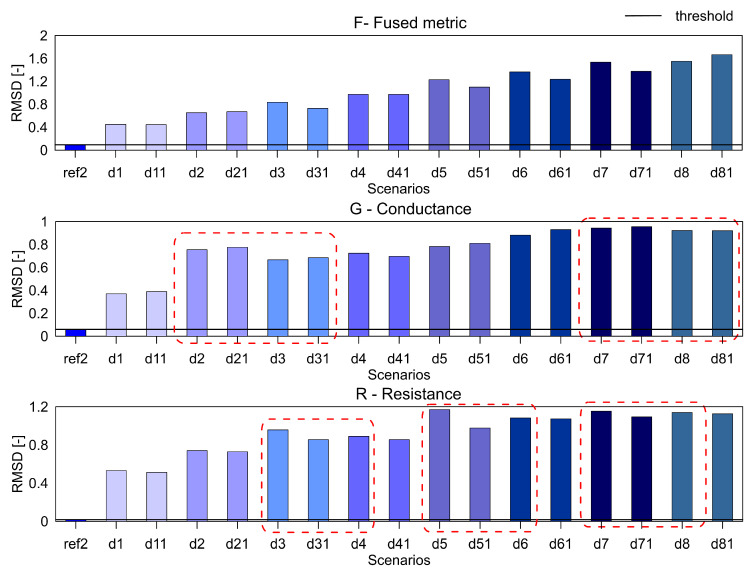
The RMSD index for the variables F, G and R for aluminium plate.

**Figure 6:**
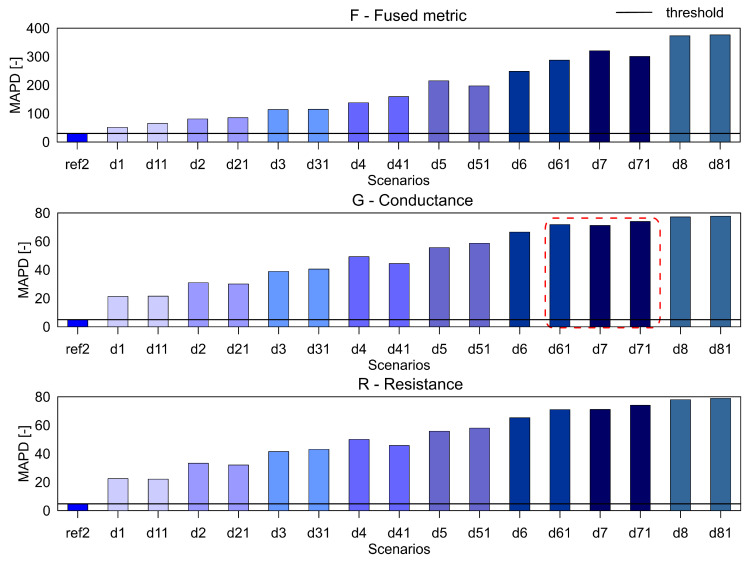
The MAPD index for the variables F, G and R for aluminium plate.

**Figure 7:**
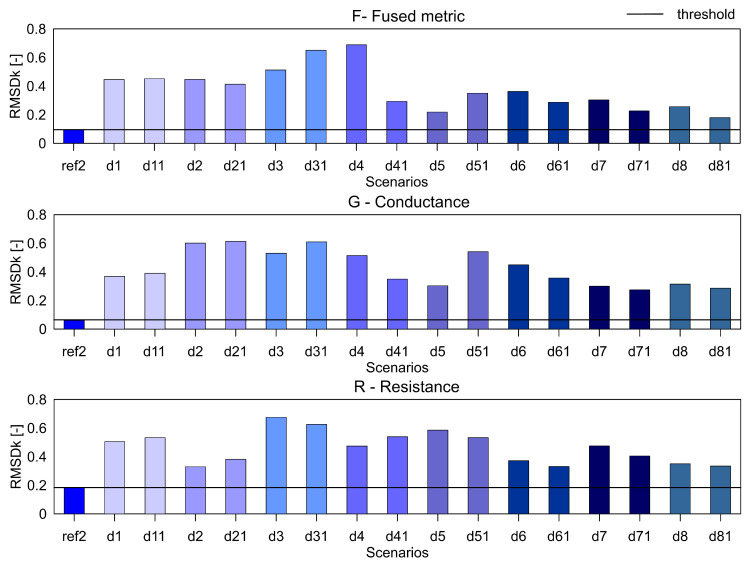
The RMSDk index for the variables F, G and R for aluminium plate.

For the aluminium plate, the measurements were carried out for the healthy scenario and eight damage scenarios (each case was measured twice). As mentioned before, three different indices, namely RMSD, MAPD and RMSDk were used for quantifying the difference in the healthy and damage cases. They are presented in Figure 5, Figure 6 and Figure 7, respectively. The threshold for the damage detection was determined based on the repeated reference measurement as it captures the inherent uncertainty in the measurements. If the index exceeds the threshold value, the damage is said to be detected.

It can be seen that the metric F for the RMSD and MAPD indices shows an increasing trend as the number of holes increases (an increase of damage severity). This trend is not seen for the G and R spectrum with notable differences highlighted by the red dotted boxes. The RMSDk compares the new measurement with the previous state, hence there is no increasing trend, but all the damage scenarios exceed the threshold value determined from the ref2 scenario.

### 4.2. Delamination Analysis of Composite Beam

**Figure 8:**
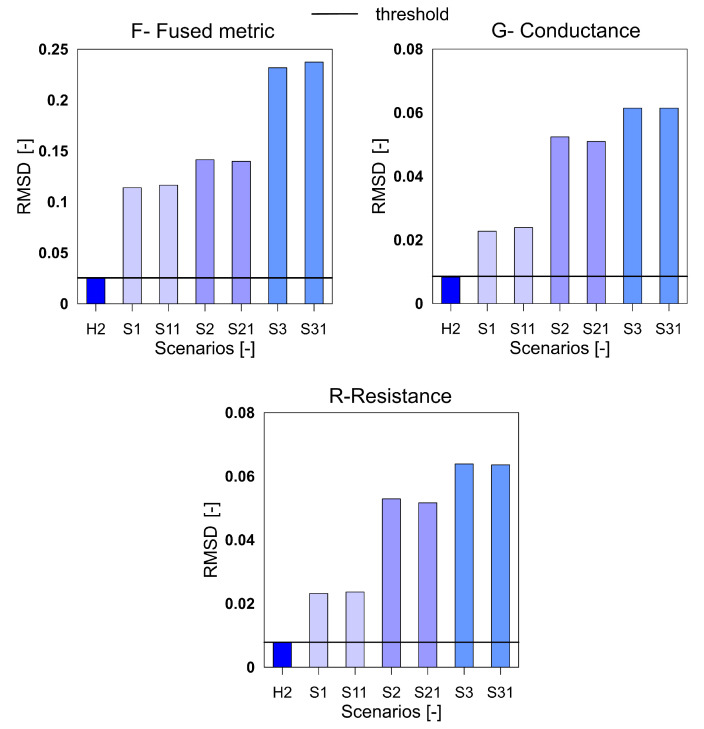
The mean absolute percentage deviation (MAPD) index for the variables F, G and R for GFRP beam.

**Figure 9:**
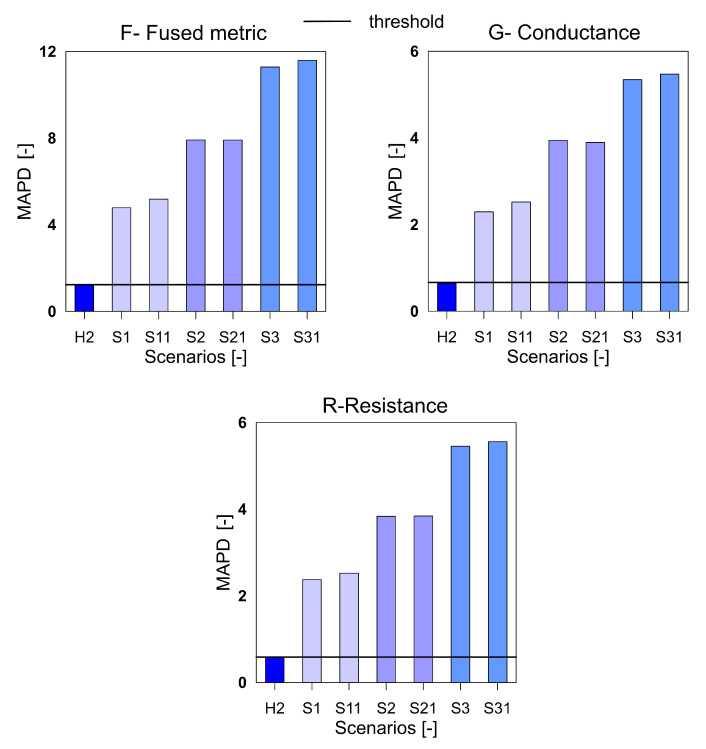
The MAPD index for the variables F, G and R for GFRP beam.

**Figure 10:**
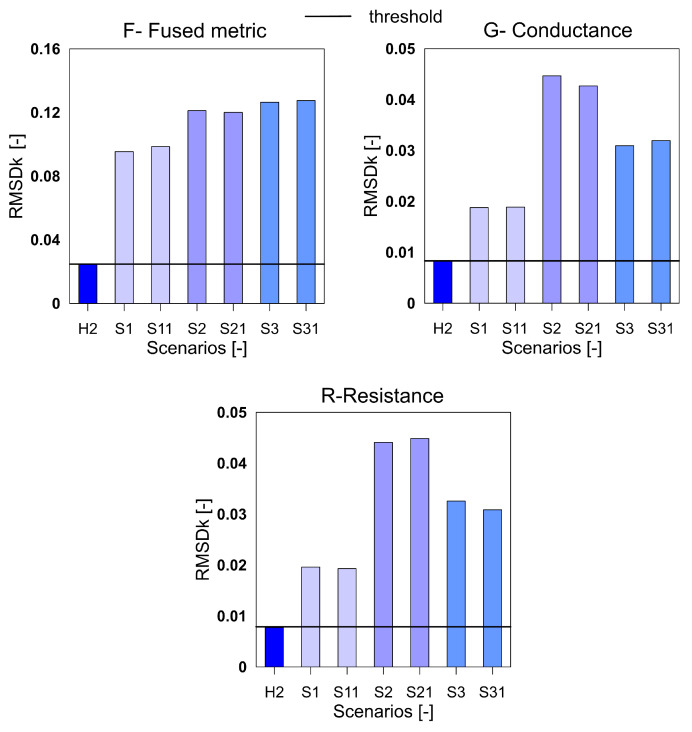
The RMSDk index for the variables F, G and R for GFRP beam.

The Figure 1 shows the rescaled spectra for the health and damage case S3. In the plot, it is clearly seen that the common peaks in the R and G spectra are amplified in the F spectra thus giving a higher contribution to the damage quantification index. Similar to the aluminum plate, the three indices for the F, G and R spectra were calculated for the measurements from the composite beam and shown in Figure 8, Figure 9 and Figure 10. It can be clearly seen that all the three indices for the three metrics clearly detect damage. Moreover, F-based RMSDk shows an increasing trend for the increase in damage severity. This is not seen for G and R based RMSDk results.

### 4.3. Impact Damage Analysis of Composite Plate

**Figure 11:**
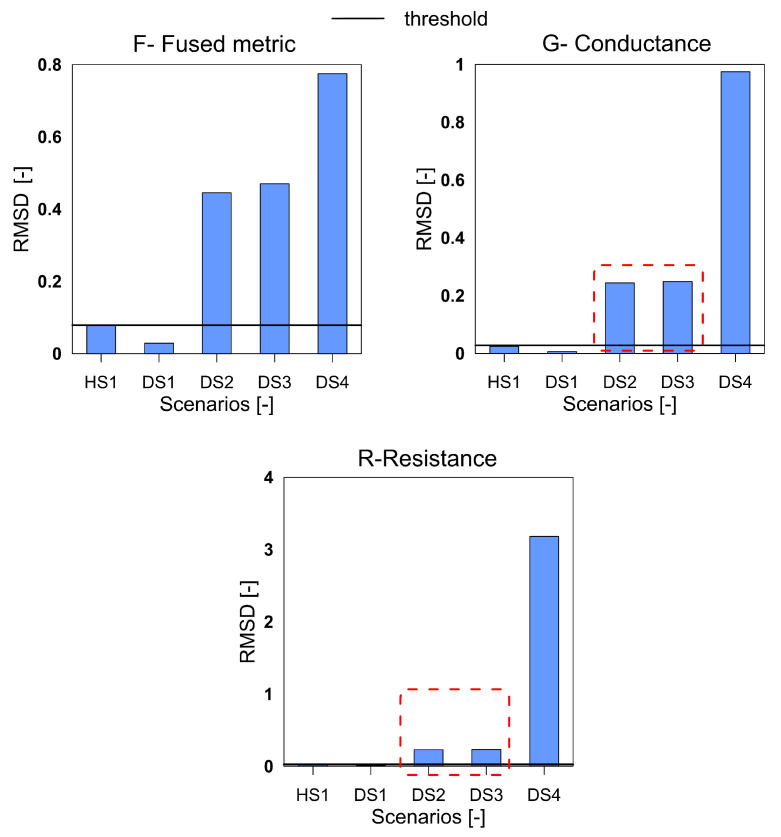
The RMSD index for the variables F, G and R for GFRP plate.

**Figure 12:**
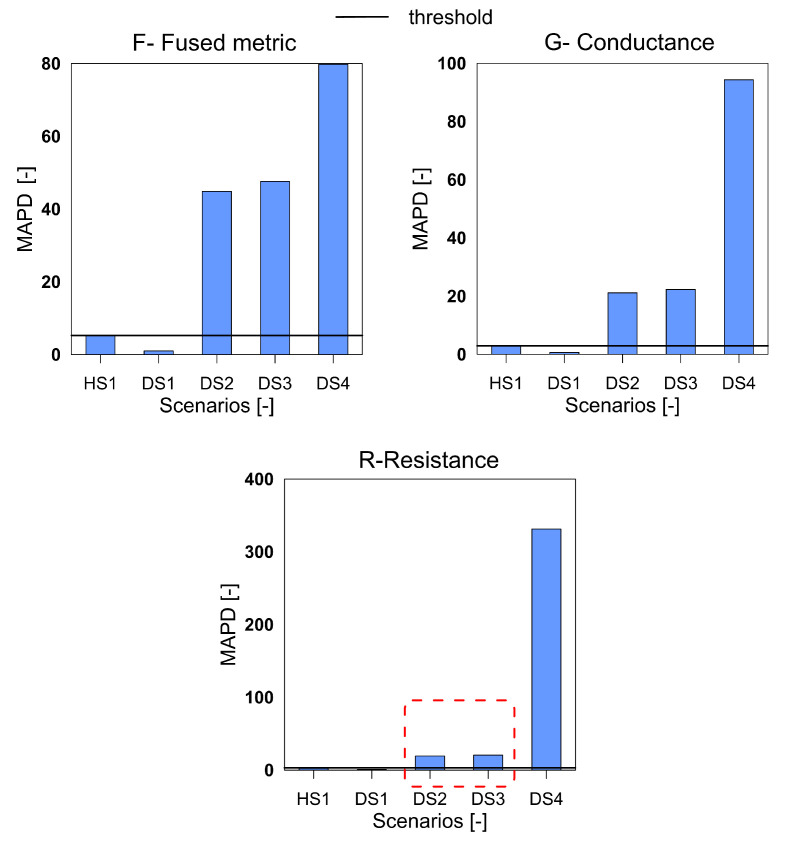
The MAPD index for the variables F, G and R for GFRP plate.

**Figure 13:**
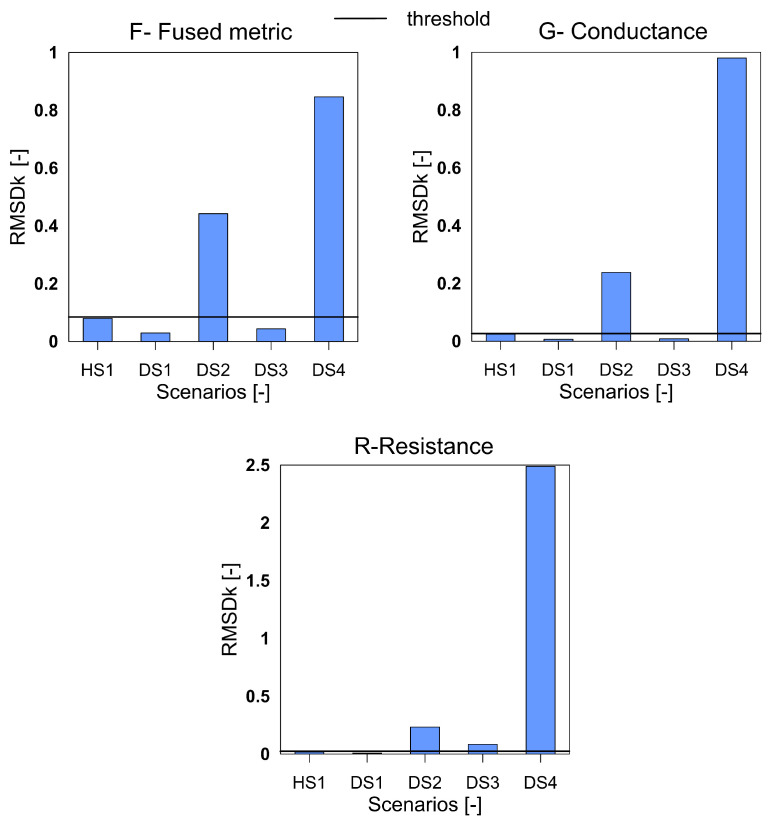
The RMSDk index for the variables F, G and R for GFRP plate.

The third structure investigated was the GFRP plate with impact-induced damage. The Figure 11, Figure 12 and Figure 13 show the three indices for F, G and R respectively.

It can be clearly seen that for all the three index with F, G, and R the damage scenario DS1 is not detected. The damage in the structure due to the first impact was very low and a significant distance away from the sensors and is understood to be the reason for missed detection. All the subsequent damage scenarios are satisfactorily detected by the three variables. A point to observe is that for the cases DS2 and DS3, the variable F shows a greater difference than the G and R variables, which points towards a higher sensitivity to differentiate between the damage of different magnitudes. Due to this enhanced sensitivity, and ability to differentiate between the damage scenarios the F metric is preferable than the G and R.

### 4.4. Summary

The comparative results of the damage index’s performance presented in Section 4.1, Section 4.2 and Section 4.3 can be summarized using Table 2. It is worth noting that, the performance of each of the variables depends on the index used for the assessment of damage. Hence the comparison of the performance of each of the variables needs to be done for the metric used for quantification.

For the aluminum plate, the G and R metrics do not show an increasing trend for the scenarios with increasing damage while the F shows a clear trend. For the MAPD index, the F and R parameters show the increasing trend while the G-based metric fails. The RMSDk index may be used for damage detection only and as such all three parameters perform satisfactorily. For the GFRP beam, all three methods show the increasing trend in the indices with an increase in the damage. and hence can be concluded that they work satisfactorily. For the GFRP plate, the smallest damage DS1, is not detected by any of the three parameters with any of the three indices. However, for the other damage scenarios, the F outperforms the G and R parameters as it can distinguish between the increased damage between scenarios DS2 and DS3. For the RMSDk metric, none of the parameters are able to detect the damage DS3 using scenario DS2 as the baseline. As a result, it can be considered that none of the parameters work for this scenario. This shortcoming of the parameters can be more attributed to the failure of the RMSDk metric than that of the parameters G, R, or F.

### 4.5. Sensitivity Studies

**Figure 14:**
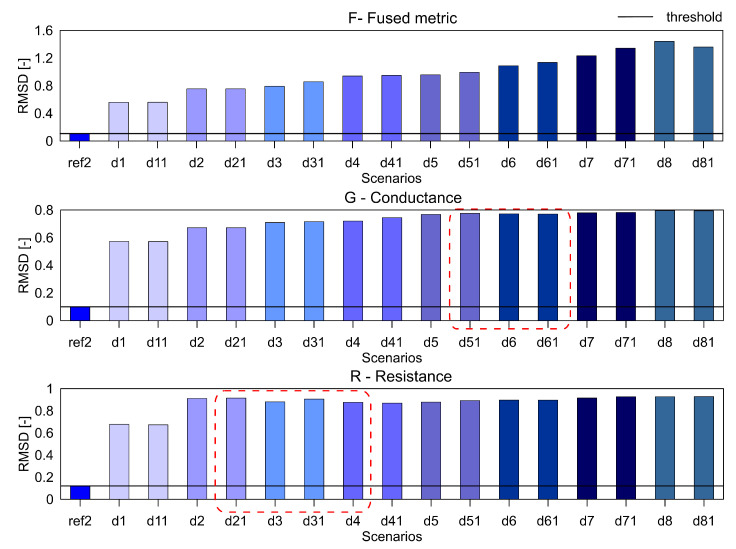
The RMSD index for the parameters F, G and R for the aluminium plate over the frequency range 100 kHz to 300 kHz.

**Figure 15:**
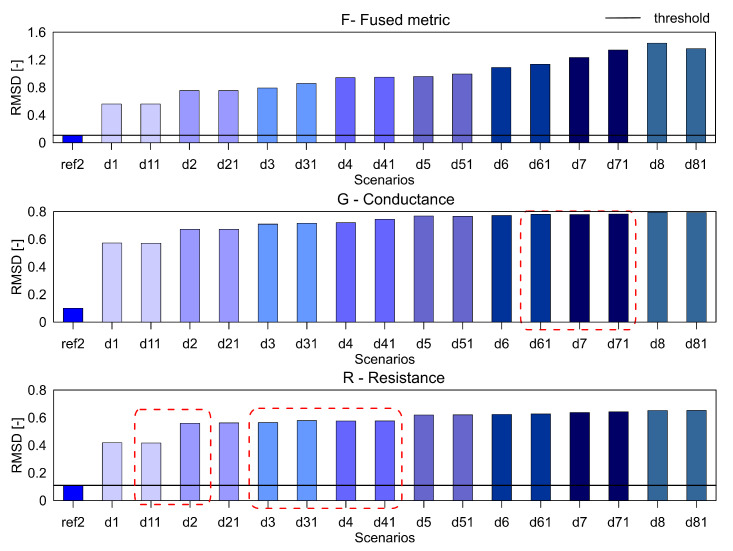
The RMSD index for the parameters F, G and R for aluminium plate over the frequency range 1 kHz to 1.3 MHz.

As mentioned earlier, the measurements for the three samples were taken over different frequency ranges, but only the frequency range from 1 kHz to 100 kHz was presented for allowing comparison. To check the robustness of the fused parameter F for the damage detection, we selected two different frequency bands namely, 100–300 kHz and 1 kHz–1.3 MHz. The results for the aluminum plate for the three parameters with the RMSD index are presented in Figure 14 and Figure 15.

In these frequency ranges, it can be seen that the F shows the expected trend of increasing values with an increase in damage severity. On the other hand, the G and R parameters do not show the increasing trend. So the fused parameter F outperforms the R and G parameters over a larger frequency range as well.

## 5. Conclusions

The RMSD, RMSDk and MAPD indices are the most popular damage indices used to detect damage in the structure using EMI technique. The motivation of the paper is to improve the performance of these indices in damage detection. The paper focused on the combination of the G and R parameters obtained from the EMI measurements to obtain the parameter F which is shown to be more sensitive for damage detection. The performance of the parameter F is compared with that of R and G for three different structures namely, an aluminum plate with sequentially introduced drilled holes, a GFRP beam with introduced delamination, and GFRP plate with introduced impact damage. In order to facilitate the comparison for different samples, the frequency range of 1 kHz to 100 kHz was used for the analysis. In addition, the robustness of the parameter F has been shown for a larger frequency range on one of the samples.

The results indicate that the F parameter is indeed more robust for the detection of damage and its increased severity. It is seen that by combining the G and R parameters the sensitivity is enhanced. This may be attributed to the smoother F spectrum achieved by the multiplication of the G and R spectra. Furthermore, the F spectra is able to detect damage over a large frequency range, which is a significant improvement over the current methods which focus on a small frequency range where the chosen parameter (G or R) is more sensitive. As this region of sensitivity is not apriori known, the application of individual parameters is challenging. The F parameter by having a larger frequency range for sensitivity allows easier application.

The authors acknowledge that this research introduces the concept of the F parameter and shows some proof of concept. It is necessary to extend the F parameter for a network of sensors and investigate its application for damage localization which is identified as the next step. In addition, the sensitivity of the parameter to ambient conditions needs to be investigated as well.

## Figures and Tables

**Table 1:** Impact damage description on GFRP plate surface.

Scenario	Description
DS1	30 J impact between P2 and P3 at a distance of 130 mm from P2
DS2	DS1 + 30 J impact between P2 and P3 at a distance of 130 mm from P2
DS3	DS2 + 30 J impact between P2 and P3 at 55 mm from P2
DS4	DS3 + 30 J impact close to P2 at a distance of 25mm from P2

**Table 2:** Comparative performance study of G, R and F variable for Al, GFRP beam and GFRP plate.

Samples	RMSD	MAPD	RMSDk
Al plate with drilled holes	F	F and R	F, G and R
GFRP beam with delamination	F, G and R	F, G and R	F, G and R
GFRP plate with impact damage	F	F and G	none
